# NAADP Activates Two-Pore Channels on T Cell Cytolytic Granules to Stimulate Exocytosis and Killing

**DOI:** 10.1016/j.cub.2012.10.035

**Published:** 2012-12-18

**Authors:** Lianne C. Davis, Anthony J. Morgan, Ji-Li Chen, Charlotte M. Snead, Duncan Bloor-Young, Eugene Shenderov, Megan N. Stanton-Humphreys, Stuart J. Conway, Grant C. Churchill, John Parrington, Vincenzo Cerundolo, Antony Galione

**Affiliations:** 1 Department of Pharmacology, University of Oxford, Mansfield Road, Oxford OX1 3QT, UK; 2 MRC Human Immunology Unit, The Weatherall Institute of Molecular Medicine, University of Oxford, John Radcliffe Hospital, Headington, Oxford OX3 9DS, UK; 3 Chemistry Research Laboratory, Department of Chemistry, University of Oxford, Mansfield Road, Oxford OX1 3TA, UK

## Abstract

A cytotoxic T lymphocyte (CTL) kills an infected or tumorigenic cell by Ca^2+^-dependent exocytosis of cytolytic granules at the immunological synapse formed between the two cells. Although inositol 1,4,5-trisphosphate (IP_3_)-mediated Ca^2+^ release from the endoplasmic reticulum activates the store-operated Ca^2+^-influx pathway that is necessary for exocytosis, it is not a sufficient stimulus [1–4]. Here we identify the Ca^2+^-mobilizing messenger nicotinic acid adenine dinucleotide phosphate (NAADP) and its recently identified molecular target, two-pore channels (TPCs) [5–7], as being important for T cell receptor signaling in CTLs. We demonstrate that cytolytic granules are not only reservoirs of cytolytic proteins but are also the acidic Ca^2+^ stores mobilized by NAADP via TPC channels on the granules themselves, so that TPCs migrate to the immunological synapse upon CTL activation. Moreover, NAADP activates TPCs to drive exocytosis in a way that is not mimicked by global Ca^2+^ signals induced by IP_3_ or ionomycin, suggesting that critical, local Ca^2+^ nanodomains around TPCs stimulate granule exocytosis. Hence, by virtue of the NAADP/TPC pathway, cytolytic granules generate Ca^2+^ signals that lead to their own exocytosis and to cell killing. This study highlights a selective role for NAADP in stimulating exocytosis crucial for immune cell function and may impact on stimulus-secretion coupling in wider cellular contexts.

## Graphical abstract

**Graphical Abstract:**

**Figure:**
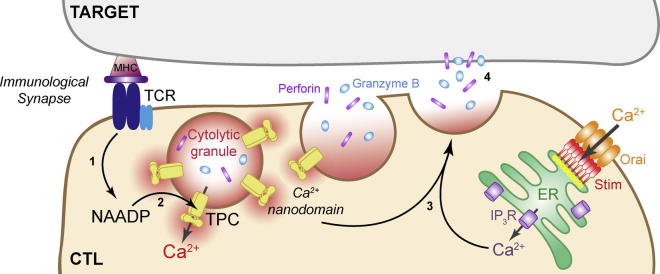

**Highlights:**

► The T cell receptor mobilizes acidic Ca^2+^ stores via NAADP/two-pore channels (TPCs) ► Exocytotic granules are themselves acidic Ca^2+^ stores decorated with TPCs ► TPCs move to the immunological synapse upon TCR activation ► NAADP stimulates exocytosis more efficiently than does IP_3_/Ca^2+^ influx alone

## Results and Discussion

The compartmentation of cell signaling pathways is important for maintaining the fidelity between extracellular stimuli and appropriate downstream responses. For Ca^2+^ signaling, not all sources (or patterns) of intracellular Ca^2+^ are equivalent, and Ca^2+^ channels can differentially couple to cellular processes [1, 8]. In T cells, the exocytosis of cytolytic factors is clearly Ca^2+^ dependent but, although Ca^2+^ influx via STIM/Orai is a necessary pathway, it is not a sufficient stimulus for exocytosis because it requires the additional activation of protein kinases [2, 9]. We investigated whether other Ca^2+^ channels couple more directly to exocytosis. All three major Ca^2+^-mobilizing second messengers—inositol 1,4,5-trisphosphate (IP_3_), cyclic ADP-ribose (cADPR), and nicotinic acid adenine dinucleotide phosphate (NAADP)—increase in concentration following T cell receptor activation [10, 11]. However, the molecular and organellar targets of NAADP are controversial in T cells [12], and the relationship between NAADP and exocytosis is unknown. To that end, we have investigated whether NAADP-dependent Ca^2+^ signaling via a recently identified molecular target, the two-pore channel (TPC) [5–7, 13], is an overlooked molecular component of T cell cytolytic granule exocytosis.

### NAADP Mobilizes Acidic Ca^2+^ Stores in Cytotoxic T Lymphocytes

First, using a pharmacological approach, we characterized the intracellular Ca^2+^ stores in a primary human cytotoxic T lymphocyte (CTL) clone and found pH-neutral as well as acidic Ca^2+^ stores. Neutral stores were revealed by ionomycin, a Ca^2+^ ionophore that can only act at neutral organelles [14], and cyclopiazonic acid (CPA), a sarcoendoplasmic reticulum Ca^2+^-ATPase inhibitor [15] (see Figures S1B–S1D available online). Similarly, agents that release Ca^2+^ from acidic organelles also produced Ca^2+^ responses, i.e., bafilomycin A1 (a V-type H^+^-ATPase inhibitor), nigericin and monensin (protonophores), and glycyl-phenylalanine 2-naphthylamide (GPN, which osmotically lyses lysosomes [16]) (Figures S1E–S1I). The presence of acidic Ca^2+^ stores is crucial because NAADP is predominantly (though not universally [12]) accepted to release Ca^2+^ from acidic (endolysosomal) Ca^2+^ stores [13, 17], thereby distinguishing it from IP_3_ and cADPR that target the neutral endoplasmic reticulum (ER).

**Figure 1:**
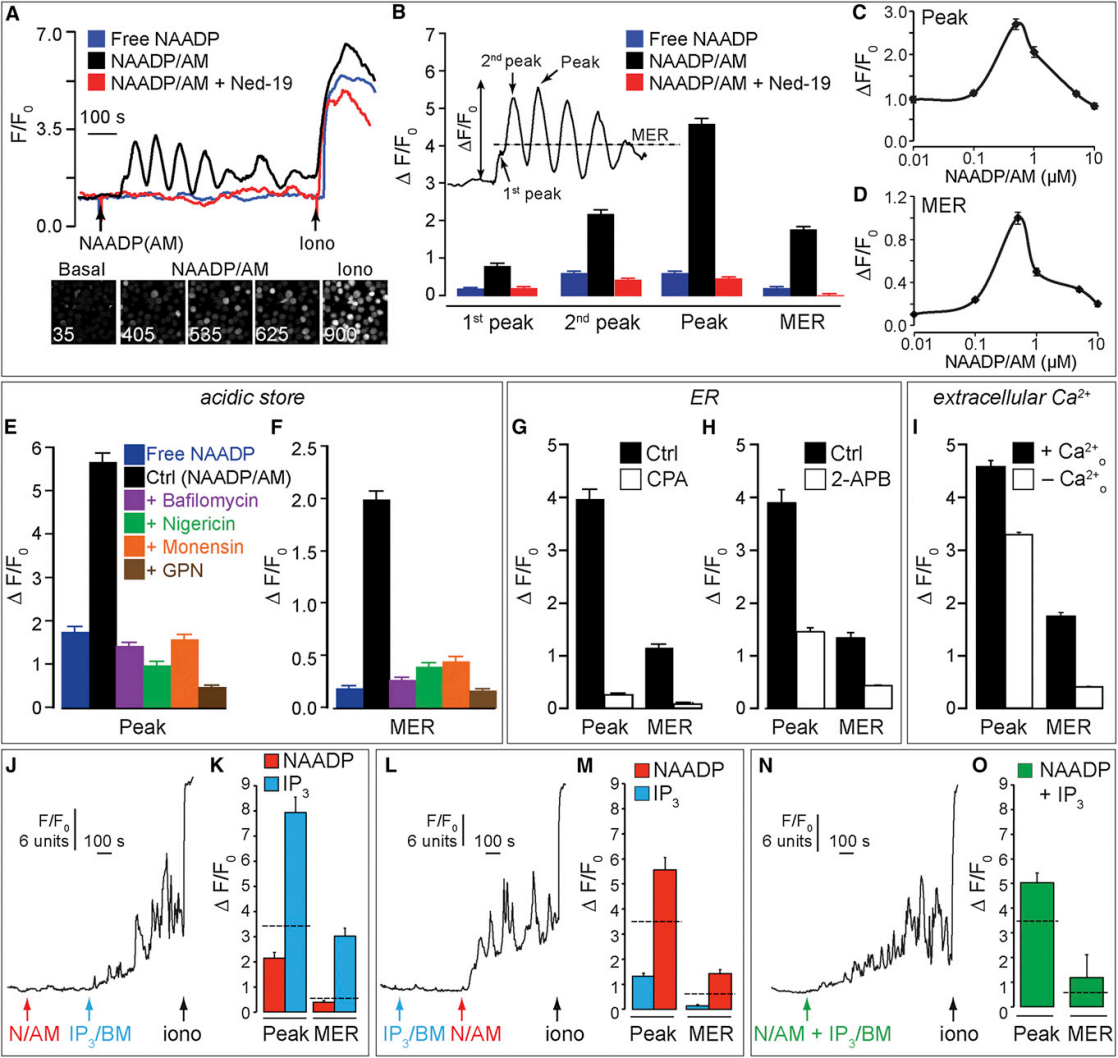
NAADP-Induced Ca^2+^ Release from Acidic Intracellular Stores in Cytotoxic T Lymphocytes (A) Single-cell Ca^2+^ traces normalized to initial fluorescence (F/F_0_). Bottom panels show fluorescence confocal images of fluo-3-loaded cytotoxic T lymphocytes (CTLs) taken at the time indicated in seconds at basal, during NAADP/AM application and upon addition of 1 μM ionomycin. NAADP/AM (2.5–10 μM) elicited Ca^2+^ oscillations, which were blocked by the NAADP antagonist *trans*-Ned-19 (10 μM). No Ca^2+^ responses were observed in response to 2.5–10 μM nonesterified (free) NAADP/DMSO. (B) Fluorescence changes (ΔF/F_0_) were calculated as shown in the inset. MER (mean elevated ratio) was defined as the mean fluorescence divided by F_0_ for the period after the addition of NAADP/AM. (C and D) NAADP/AM-induced Ca^2+^ release displayed a “bell-shaped” concentration-response curve, characteristic of this second messenger. (E and F) Ca^2+^ signals to 2.5–10 μM NAADP/AM were inhibited by preincubation with 1 μM bafilomycin A1, 10 μM nigericin, 1 μM monensin, or 50 μM GPN. n = 383−1,030 cells. (G and H) CTLs were treated with 10 μM CPA in Ca^2+^-free buffer (G) or 2 μM 2-APB (H) prior to application of 10 μM NAADP/AM. n = 81–178 cells. (I) Ca^2+^ signals with 2.5–10 μM NAADP/AM in 0.5 mM extracellular Ca^2+^ (+Ca^2+^_o_) or Ca^2+^-free medium containing 100 μM EGTA (–Ca^2+^_o_). n = 730–1,030 cells. (J–O) Traces from single cells (J, L, and N) and Ca^2+^ changes (K, M, and O) (n = 66–99 cells) upon addition of 1 μM NAADP/AM followed by 5 μM IP_3_/BM (J and K) or vice versa (L and M). Cells responded to ionomycin (1 μM) at the end of the recording. The dotted line represents the sum of NAADP/AM alone and IP_3_/BM alone responses. In (N) and (O), 5 μM IP_3_/BM and 1 μM NAADP/AM were added simultaneously. ^∗∗∗^p < 0.001 versus Ctrl. All phases of the Ca^2+^ signal for free NAADP versus NAADP/AM + inhibitor are not significantly different. Error bars are mean ± SEM. See also Figure S1.

To test whether NAADP could evoke Ca^2+^ signals in CTLs, we indirectly introduced NAADP into the cytosol by bath application of a membrane-permeant ester precursor (NAADP/AM) that is converted to NAADP by intracellular esterases. NAADP/AM elicited Ca^2+^ oscillations, with the initial spike commonly comprising distinct first and second phases (Figures 1A and 1B); oscillations were blocked by the NAADP antagonist *trans*-Ned-19 (Figures 1A and 1B) [18]. The response to NAADP/AM was not an artifact of contaminating nonesterified (free) NAADP acting at plasma membrane receptors because equimolar extracellular NAADP was without effect (Figures 1A and 1B). Strikingly, NAADP/AM-induced Ca^2+^ release displayed a “bell-shaped” concentration-response curve (Figures 1C and 1D), which is a hallmark of this second messenger [19]. Repeating experiments in Ca^2+^-free medium (to eliminate Ca^2+^ influx), NAADP/AM still caused Ca^2+^ oscillations, thus confirming intracellular stores as the primary Ca^2+^ source (Figures S1J–S1N). That NAADP required acidic Ca^2+^ stores was confirmed by inhibition of oscillations by bafilomycin A1, nigericin, monensin, or GPN (Figures 1E and 1F). Given that Ned-19 acts upon non-ER Ca^2+^ channels [18], this provides compelling evidence that NAADP targets acidic organelles in primary CTLs, contrasting with results in a Jurkat cell line [20].

The long-standing “trigger hypothesis” or two-pool model [13, 21] describes NAADP as a provider of an initial “trigger” bolus of Ca^2+^ that is subsequently amplified by Ca^2+^ release from the ER by virtue of the Ca^2+^ sensitivity of the IP_3_ receptor (IP_3_R) or ryanodine receptor (RyR), so-called Ca^2+^-induced Ca^2+^ release (CICR). We tested for the coinvolvement of the ER, first by depleting the ER with the Ca^2+^-ATPase inhibitor CPA, which did indeed abrogate NAADP/AM responses (Figures 1G, S2C, and S1D). Also consistent with ER involvement is the fact that NAADP/AM stimulated Ca^2+^ influx (Figure 1I), a natural consequence of ER depletion and recruitment of the dominant store-regulated Ca^2+^ entry pathway in T cells.

**Figure 2:**
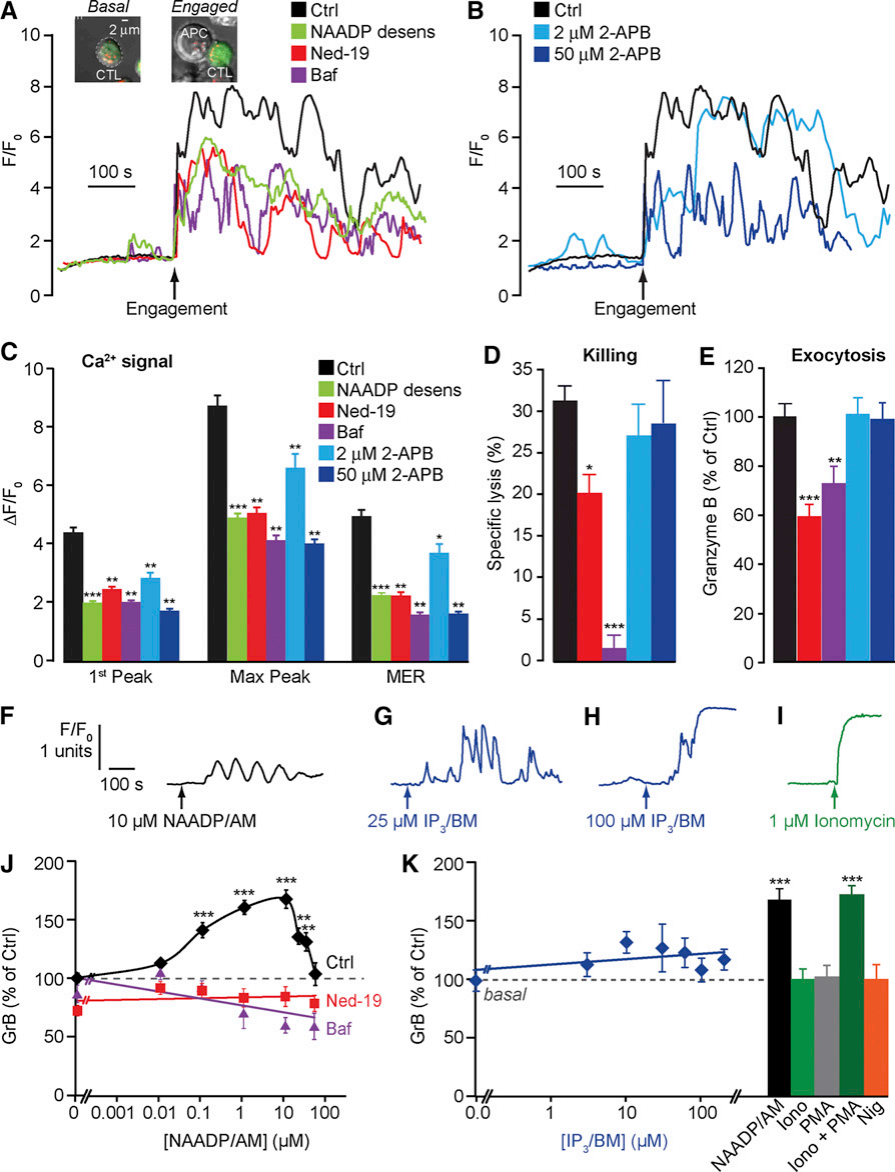
NAADP Is Involved in Physiological T Cell Receptor Activation (A–C) Ca^2+^ signals in CTLs upon engagement with antigen-presenting cells (APCs) loaded with 100 nM ESO 9C peptide. CTLs were incubated with 0.1% DMSO (Ctrl), 10 μM *trans*-Ned-19, 1 μM bafilomycin A1, or 2 μM or 50 μM 2-APB prior to engagement and throughout the experiment. (A and B) Single-cell Ca^2+^ traces. Inset: confocal images of CTL loaded with fluo-3 and Lysotracker Red to label acidic organelles before (basal) and after engagement with APCs. (C) Peak Ca^2+^ changes (ΔF/F_0_) and MER (mean value of fluorescence throughout the postengagement period) as normalized to F_0_. n = 130–239 cells. (D) Lysis of target cells pulsed with ESO 9C peptide by CTL (measured by release of ^51^Cr after 2 hr) under conditions as in (A)–(C). n = 6. (E) Granzyme B secretion by CTLs 15 min after antigen presentation, under conditions as in (A)–(C) and measured by ELISA. n = 6–15. (F–I) Ca^2+^ signal upon addition of NAADP/AM (F), IP_3_/BM (G and H), and ionomycin (I) to fluo-3-loaded CTL. (J and K) In the absence of antigen presentation, CTLs were stimulated with NAADP/AM in the presence of 0.1% DMSO (Ctrl), 10 μM *trans*-Ned-19, or 1 μM bafilomycin A1 (J); CTLs were treated with IP_3_/BM, 10 μM NAADP/AM, 10 μM nigericin, 1 μM ionomycin, 50 nM PMA, or a combination of 1 μM ionomycin and 50 nM PMA (K). After 30 min, supernatants were assayed for granzyme B by ELISA (n = 3). ^∗∗∗^p < 0.001, ^∗∗^p < 0.01, ^∗^p < 0.05. Error bars are mean ± SEM. See also Figure S2.

Given that IP_3_R1–3 and RyR1 were all detected by PCR in these primary T cells (Figures S3A and S3B), we tested which ER channel family was functionally important. We found no evidence of functional RyRs, either with or without NAADP/AM (Figures S1O–S1Q), conceivably due to a low RyR abundance [22]. In contrast, using a cell-permeant analog of the major Ca^2+^-mobilizing second messenger IP_3_ (IP_3_/BM), IP_3_Rs were demonstrably active (Figures 1J–1O, 2G, 2H, S2A, and S2B), and complementary pieces of evidence suggested that they contribute to NAADP-evoked responses: first, IP_3_R blockade with 2-APB (2-aminoxydiphenylborate) (Figures S2A and S2B) [23] profoundly reduced NAADP/AM-stimulated Ca^2+^ oscillations (Figure 1H). Conversely, costimulating IP_3_Rs enhanced NAADP/AM-induced Ca^2+^ release (Figures 1J–1O), and in a synergistic (greater than additive) manner that depended upon the order of addition, with the greatest effect occurring when NAADP was added first. Together, these data are consistent with the trigger hypothesis whereby NAADP provides the trigger Ca^2+^ from acidic stores that is subsequently amplified by IP_3_Rs on the ER [13, 19].

### The T Cell Receptor Recruits the NAADP Pathway

Having defined NAADP signaling in CTLs, we determined whether it was involved during physiological T cell receptor (TCR) activation. CTLs were stimulated upon engagement with peptide-loaded antigen-presenting cells (APCs), which resulted in the expected two phases of Ca^2+^ increase: an immediate spike (Ca^2+^ release from intracellular stores), followed by a slower, sustained increase or Ca^2+^ oscillations (dependent on Ca^2+^ influx; Figures 2A and 2B [24]). Acidic Ca^2+^ stores and NAADP did indeed contribute to TCR-induced Ca^2+^ signals as judged by the effect of inhibitors of the NAADP pathway: Ned-19 or bafilomycin A1 and NAADP self-desensitization profoundly reduced the amplitudes of the Ca^2+^ peaks as well as the mean fluorescence (an index of how sustained the response is; Figures 2A and 2C). In addition to NAADP, IP_3_Rs and Ca^2+^ influx were also recruited as expected (and required; Figure 2B): 2-APB attenuated Ca^2+^ oscillations at low (2 μM) and high (50 μM) concentrations, respectively (Figure 2C). Together, these results highlight a physiological role for NAADP-induced Ca^2+^ release that interleaves with Ca^2+^-induced Ca^2+^ release (IP_3_Rs) and Ca^2+^ influx.

### NAADP Stimulates Cytolytic Granule Exocytosis

We next asked whether NAADP/acidic stores were important for exocytosis and target cell killing. CTL-dependent killing of target cells can be effected by two processes: (1) the Ca^2+^-dependent vectorial exocytosis of cytolytic proteins granzyme B and perforin from CTL cytolytic granules (a type of secretory lysosome [25]), and (2) the cell-surface expression of the death receptor Fas ligand [26]. A link between Ca^2+^ influx and cytolytic granule exocytosis is well established [3], but a role for acidic Ca^2+^ stores in driving secretion is less understood. Therefore, we measured granzyme B secretion at early times (15–30 min) after APC engagement. Testing for NAADP and acidic Ca^2+^ stores, we found that Ned-19 or bafilomycin A1 substantially inhibited granzyme B exocytosis (Figure 2E) and this translated into an inhibition of cell killing (Figure 2D). Importantly, this was not merely a consequence of a reduced global Ca^2+^ signal, because 2 μM and 50 μM 2-APB also lowered the TCR Ca^2+^ signal to the same extent (Figure 2C) and yet secretion and cell killing were unaffected (Figures 2D and 2E). Bafilomycin A1 had a disproportionate effect upon killing compared to exocytosis, which is probably due to an additional effect upon the target cells themselves: bafilomycin A1 (and other V-ATPase inhibitors) have been shown to profoundly block the perforin-dependent cytotoxicity mediated by CTLs [27]. In keeping with the Ca^2+^ data, 10 μM ryanodine did not affect TCR-stimulated granzyme B release (93% ± 8% of control, n = 8), excluding a role for RyRs in exocytosis. In summary, these data implicate NAADP in TCR-stimulated exocytosis.

We directly tested whether NAADP could stimulate exocytosis, even in the absence of TCR activation. Remarkably, application of NAADP/AM per se was sufficient to evoke granzyme B secretion in a Ned-19- and bafilomycin A1-sensitive manner (Figure 2J). Moreover, the hallmark bell-shaped NAADP/AM concentration-response curve previously observed with Ca^2+^ signals (Figures 1C and 1D) was preserved with granzyme B exocytosis (Figure 2J). Together, the data indicate that NAADP can and does drive exocytosis.

If NAADP simply acts by elevating global Ca^2+^, then any experimental manipulation that mimics this should stimulate exocytosis. Therefore, we raised Ca^2+^ in other ways and assessed secretion. IP_3_/BM evoked robust Ca^2+^ signals in CTLs, and in a classic pattern dependent on the stimulus intensity: lower concentrations of IP_3_/BM stimulated oscillations, whereas higher concentrations gave a strong peak-and-plateau Ca^2+^ pattern (indicative of Ca^2+^ release and influx through Orai/STIM activation; Figures 2G and 2H). Although IP_3_-induced Ca^2+^ signals mimic those seen following stimulation by physiological TCR engagement [24] or NAADP/AM (Figures 2A and 2F), granzyme B secretion was not observed (Figure 2K). Likewise, the Ca^2+^ ionophore ionomycin produced a strong Ca^2+^ signal (Figure 2I) but failed to evoke exocytosis (unless combined with phorbol esters to activate PKC/ERK [4, 26]; Figure 2K). Interestingly, releasing Ca^2+^ from the acidic Ca^2+^ store using nigericin (Figure S1E) was not sufficient to evoke exocytosis, reinforcing the unique properties of NAADP (Figure 2K). That is, in the absence of TCR activation, only NAADP-induced Ca^2+^ release coupled efficiently to exocytosis, thereby reinforcing the different roles of second messengers, i.e., NAADP and IP_3_ [13, 19].

What underlies this differential coupling? A major difference between NAADP and the other agents is that NAADP ultimately recruits three Ca^2+^ sources: acidic Ca^2+^ stores (Figures 1E and 1F), the ER (Figures 1G and 1H), and, consequently, extracellular Ca^2+^ (Figure 1I). In contrast, IP_3_ and ionomycin only share the latter two (neither mobilizes acidic Ca^2+^ stores [14]). This suggests that Ca^2+^ release from acidic stores by NAADP is a crucial permissive factor for exocytosis. Indeed, although Ca^2+^ influx via Orai/STIM is crucial for lymphocyte function, it cannot drive exocytosis without the pharmacological activation of protein kinases [2, 9]. Therefore, NAADP is the first Ca^2+^ pathway demonstrated to drive exocytosis without additional stimuli. Given that the global Ca^2+^ signal is a poor predictor of exocytosis, we hypothesize that local Ca^2+^ nanodomains around the acidic Ca^2+^ stores are what distinguishes NAADP from the other stimuli. This underscores the importance of where the Ca^2+^ is released, i.e., local versus global Ca^2+^ responses, and so we turned to identifying the molecular target of NAADP, and thereby its subcellular locale.

### NAADP-Dependent Ca^2+^ Signals during TCR Activation Require Two-Pore Channels

**Figure 3:**
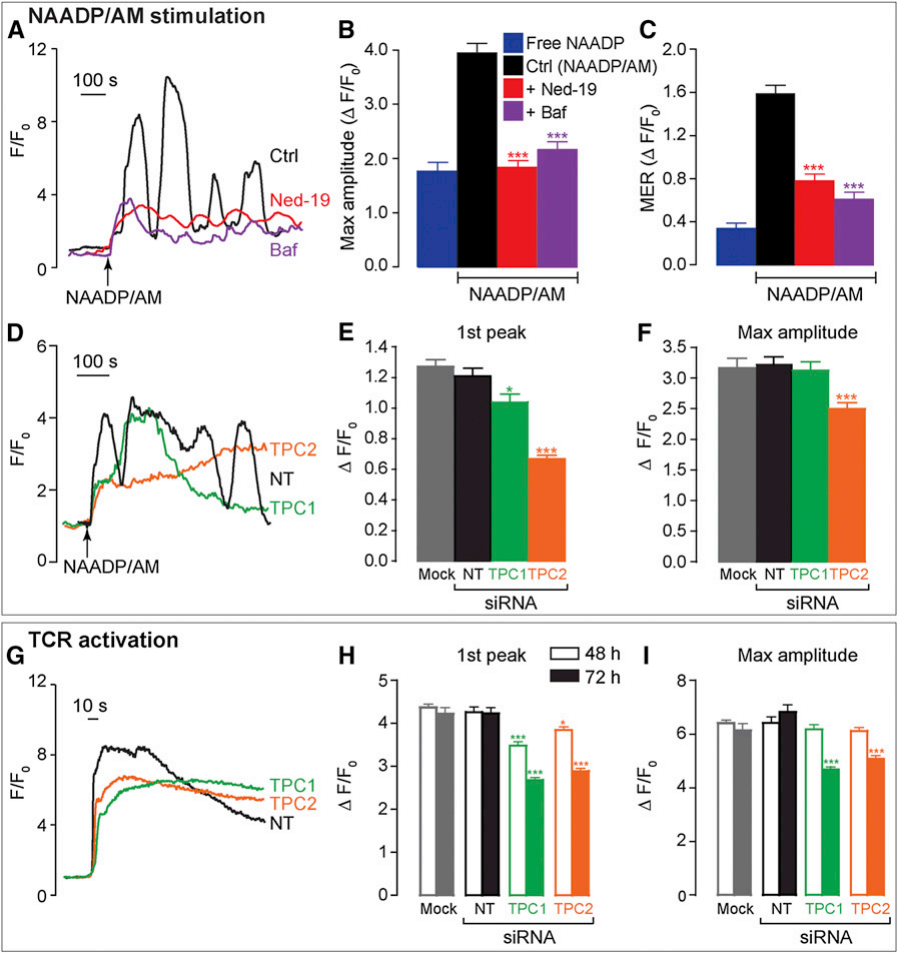
TPCs Are Necessary for TCR-Triggered Ca^2+^ Release via NAADP (A–C) Ca^2+^ oscillations in Jurkat 1G4 cells induced by 2.5–10 μM NAADP/AM were inhibited by 10 μM *trans*-Ned-19 or 1 μM bafilomycin A1. Single-cell Ca^2+^ traces (A) and summary of maximum peak Ca^2+^ changes and MER (B and C) are shown. n = 88–103 cells. (D–I) Jurkat 1G4 cells were nucleofected with 100 pmol siRNA (nontargeting [NT], TPC1 and TPC2) or without siRNA (Mock) and incubated for 72 hr (D–G) or 48–72 hr (H and I). siRNA-treated cells were stimulated with 10 μM NAADP/AM (D–F) or by contact with 10 μg/ml anti-human CD3-coated coverslips (G–I). Single-cell Ca^2+^ traces (D and G) and summary of peak Ca^2+^ changes (E–F and H–I) are shown for n = 213–383 cells from six independent siRNA experiments. Error bars are mean ± SEM. See also Figure S3.

Recently, the major ion channel target for NAADP has been identified as members of the TPC family that appropriately reside on acidic compartments of the endolysosomal system in various mammalian cell types [5–7]. To address the importance of TPCs in TCR activation, we used Jurkat cells transduced with the NY-ESO-1_157–165_-HLA A2-restricted TCR 1G4 [25], hereafter referred to as Jurkat 1G4 cells, because they are amenable to genetic manipulation and express the 1G4 TCR, which has the same specificity as the TCR expressed by the 4D8 CD8+ T cell clone used above. We established that Jurkat 1G4 cells mirrored the 4D8 T cell clone in several key aspects: first, each cell type expresses both TPC1 and TPC2 isoforms as judged by RT-PCR (Figures S3C and S3F), and second, NAADP/AM elicited Ca^2+^ responses in Jurkat 1G4 cells that were sensitive to Ned-19 and bafilomycin A1 (Figures 3A–3C), consistent with acidic Ca^2+^ store involvement [22, 28].

The functional role of TPCs in NAADP-induced Ca^2+^ release was tested by suppressing TPC1 and TPC2 expression with small interfering RNA (siRNA) knockdown (assessed by RT-qPCR and western blot; Figures S3G–S3I). Consequently, NAADP/AM-evoked Ca^2+^ release was reduced by TPC siRNAs, with a more pronounced inhibition of Ca^2+^ signals seen with TPC2 than with TPC1 siRNA (Figures 3D–3F and S3J). Statistical analysis suggested that only a subpopulation of cells were affected by siRNA (Figure S3J), presumably a function of the transfection efficiency (although we could not empirically identify which cells were transfected). The Ca^2+^ signal was significantly reduced in amplitude (by 30%–54%)—a partial effect consistent with incomplete TPC knockdown (Figures S3G–S3I)—and underscored the importance of TPCs for NAADP action. Because NAADP is important for TCR signaling (Figure 2), it follows that TPCs should likewise be required, and indeed, TCR-induced Ca^2+^ signals were reduced with either TPC1 or TPC2 siRNA (maximal inhibition 44% for each isoform; Figures 3G–3I and S3K). Incidentally, RyRs were absent in Jurkat 1G4 cells (Figure S3E, as with other Jurkat T cell clones [22, 28]), confirming that RyRs are not obligatory for NAADP action, though they have been proposed as NAADP receptors by others [12, 13]. The data affirm both TPC isoforms as necessary components of NAADP-dependent Ca^2+^ signals during TCR activation, and so CTLs join the emerging list of cells that utilize the NAADP/TPC couple [13, 16].

### TPCs on Exocytotic Granules Translocate to the Immunological Synapse

Thus far, we had not determined precisely which acidic vesicle class (or classes) the NAADP/TPC axis was associated with. To that end, we tagged TPC1 and TPC2 with fluorescent proteins and compared their distribution in Jurkat 1G4 cells with other acidic vesicle markers. Both TPC1 (Figures S4A–S4C) and TPC2 (Figures S4D–S4F) colocalized with two markers of the cytolytic granules themselves (granzyme B and LAMP-1), although the TPC1 overlap was slightly lower than that for TPC2 (distribution differences between the two isoforms are a recurring theme [13]). This overlap was not peculiar to Jurkat 1G4 cells, because we observed a similar pattern in primary CTLs: using immunofluorescence, endogenous TPC2 extensively colocalized with granzyme B and LAMP-1 (Figures S4G–S4I). The specificity of TPC2 antibody labeling was verified by preblocking with the immunogenic peptide, which reduced labeling by 87.4% ± 0.4%. Unfortunately, we were unable to obtain specific staining with anti-TPC1 antibodies. NAADP therefore targets the exocytotic vesicles themselves.

That TPCs are found on cytolytic granules agrees with the NAADP acidic store pharmacology (Figures 1E and 1F) because cytolytic granules are the acidic, secretory lysosomes of CTL and natural killer (NK) cells [25, 29]. Indeed, TPC2 was identified in the proteome of NK cell secretory lysosome membranes [30], and such a localization provides the appropriate architecture for NAADP-evoked local Ca^2+^ domains that selectively couple to exocytosis. That secretory vesicles act as Ca^2+^ stores has some precedents [13, 31, 32].

**Figure 4:**
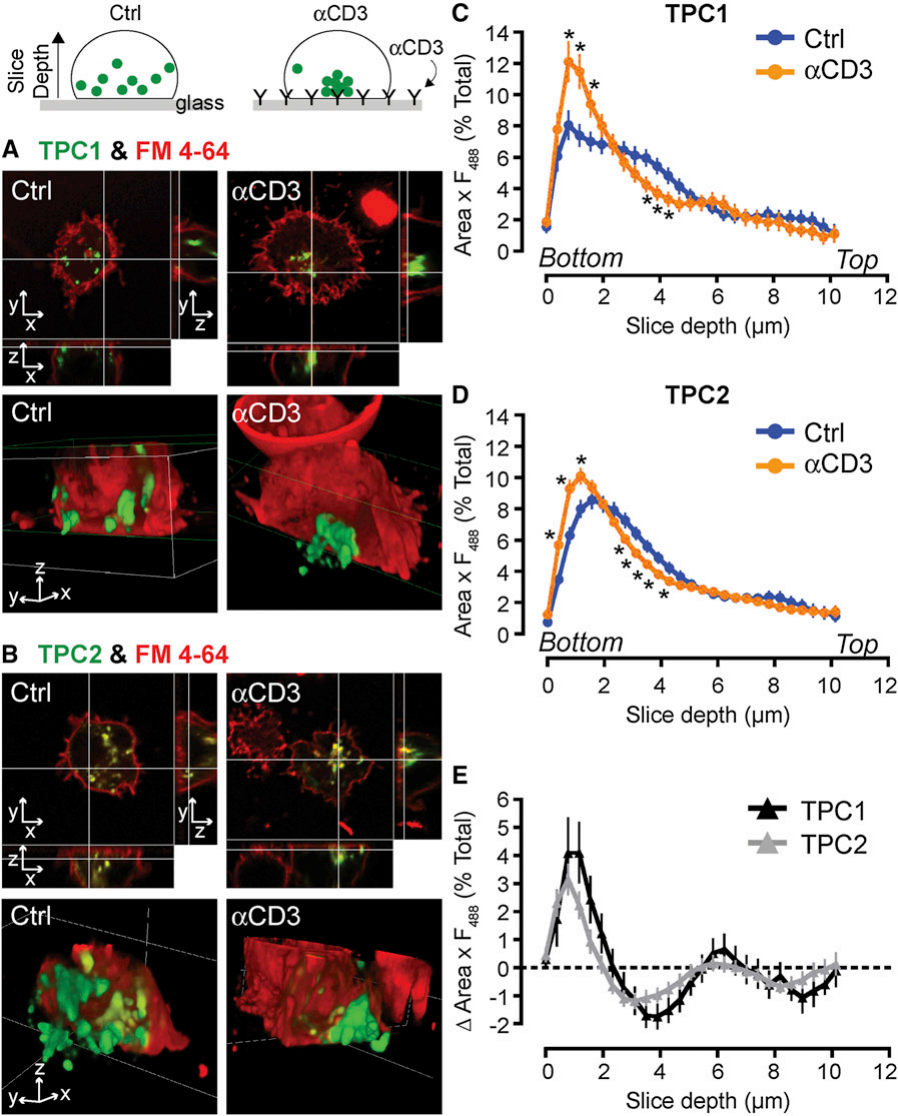
TPCs Translocate to an Immunological Synapse upon Stimulation with Anti-CD3 eYFP-tagged HsTPC1 and HsTPC2 (green) were expressed in Jurkat 1G4 cells and added to glass coverslips coated with poly-L-lysine or 10 μg/ml anti-human CD3 antibody mounted in an imaging chamber, as depicted in the cartoon. Cells were labeled with FM4-64 (red) to delineate the plasma membrane edge of the cell. (A and B) Confocal images of cells attached to poly-L-lysine (Ctrl; left-hand side) or engaged with anti-CD3 antibody (αCD3; right-hand side) showing z stacks through x and y axes (top images) and 3D reconstruction of the z stack (bottom images). TPC localization was assessed through the z stack as a product its area and its fluorescence, as depicted in (C)–(E). A slice depth of 0 indicates the bottom of the cell (i.e., the contact zone between cell and the coverslip). (C and D) TPC1 (C) and TPC2 (D) localization significantly alters from being present deeper in the cell (slice depth 3–5 μm) toward αCD3, i.e., the bottom of the cells (slice depth 0–2 μm) compared to control. (E) Comparison of TPC1 and TPC2 movement in cells engaged with anti-CD3. There was no significant difference in the migration of the two isoforms. TPC1 n = 32–43; TPC2 n = 52–69. Error bars are mean ± SEM. See also Figure S4.

An important characteristic of cytolytic granules is that they rapidly polarize along microtubules toward the immunological synapse (IS) when the TCR is activated (Figures S4J and S4K) [25]. Because TPC1 and TPC2 are expressed on these granules, they should also move to the IS. CTLs, including Jurkat clones [33], can form a pseudo-IS with anti-CD3-coated glass coverslips, and so we examined the 3D distribution of fluorescently tagged TPCs in adherent cells in the presence or absence of antibody coating. Under control conditions without antibody, TPC puncta were distributed throughout the cell (Figures 4A–4D). However, when anti-CD3 was present at the contact surface, a pronounced translocation of both TPC1 and TPC2 from deeper regions of the cell to the contact surface was observed (Figures 4A–4E). Figure 4E shows that TPC1 and TPC2 translocate to the same extent, and their clustering may contribute to the high Ca^2+^ domains required for lytic granule exocytosis [3]. Moreover, immunolabeled TPC2 in primary CTLs shows endogenous polarization to the IS within a primary CTL:target cell conjugate (Figures S4L and S4M). This is the first report of a directed movement and clustering of TPCs during a specific response.

### Conclusion

In summary, TCR activation recruits NAADP to activate target TPC channels resident on the exocytotic granules themselves, and TPCs consequently translocate toward the immunological synapse. Therefore, these granules store and release the Ca^2+^ for their own exocytosis and deliver Ca^2+^ in an “autocrine” fashion via TPCs, presumably acting in local perigranular Ca^2+^ nanodomains [8]. In the absence of TCR activation, other Ca^2+^ signaling pathways, e.g., IP_3_, fail to mimic NAADP. Although the Orai1-STIM1 complex (CRAC channels) makes a substantial contribution to CTL Ca^2+^ signals [3], activation of Ca^2+^ influx per se is insufficient to drive exocytosis, requiring the additional presence of protein kinase activators [2, 9]; the NAADP/TPC pathway is therefore all the more remarkable for being able to drive exocytosis. Clearly, CRAC channels are important for sustaining exocytosis [1, 3], but we propose that local NAADP/acidic store/TPC signaling is an additional important component.

## Supplemental Information

Document S1. Figures S1–S4 and Supplemental Experimental Procedures
