# Supplementary material for: NAADP Activates Two-Pore Channels on T Cell Cytolytic Granules to Stimulate Exocytosis and Killing
Source: Curr Biol. 2012 Dec 18;22(24):2331–7. doi: 10.1016/j.cub.2012.10.035 (PMC3525857; doi:10.1016/j.cub.2012.10.035)
Supplement: Document S1. Figures S1–S4 and Supplemental Experimental Procedures [file mmc1.pdf]

Current Biology, Volume *22*

Supplemental Information

NAADP Activates Two-Pore Channels on T Cell Cytolytic Granules to Stimulate Exocytosis and Killing

Lianne C. Davis, Anthony J. Morgan, Ji-Li Chen, Charlotte M. Snead, Duncan Bloor-Young, Eugene Shenderov, Megan N. Stanton-Humphreys, Stuart J. Conway, Grant C. Churchill, John Parrington, Vincenzo Cerundolo, and Antony Galione

Supplemental Inventory

Supplemental Data

Figure S1, related to Figure 1

Ca^{2+} signals in CTL upon stimulation with NAADP/AM.

Figure S2, related to Figure 2

2-APB blocked IP_3Rs and CPA emptied ER Ca^{2+} stores.

Figure S3, related to Figure 3

TPC expression is reduced by siRNA.

Figure S4, related to Figure 4

TPCs are localized on exocytotic (cytolytic) granules.

Supplemental Experimental Procedures

Cells and reagents
Intracellular Ca^{2+} measurements
Human Granzyme B ELISA
^{51}Cr-release assay
Reverse transcription and PCR amplification (RT-PCR)
Restriction enzyme analysis of RT-PCR products
Nucleofection of Jurkat 1G4 with plasmid DNA and siRNA
RT-qPCR
Immunoblotting
Immunolocalization
TPC Translocation to the IS
Data analysis

Supplemental References

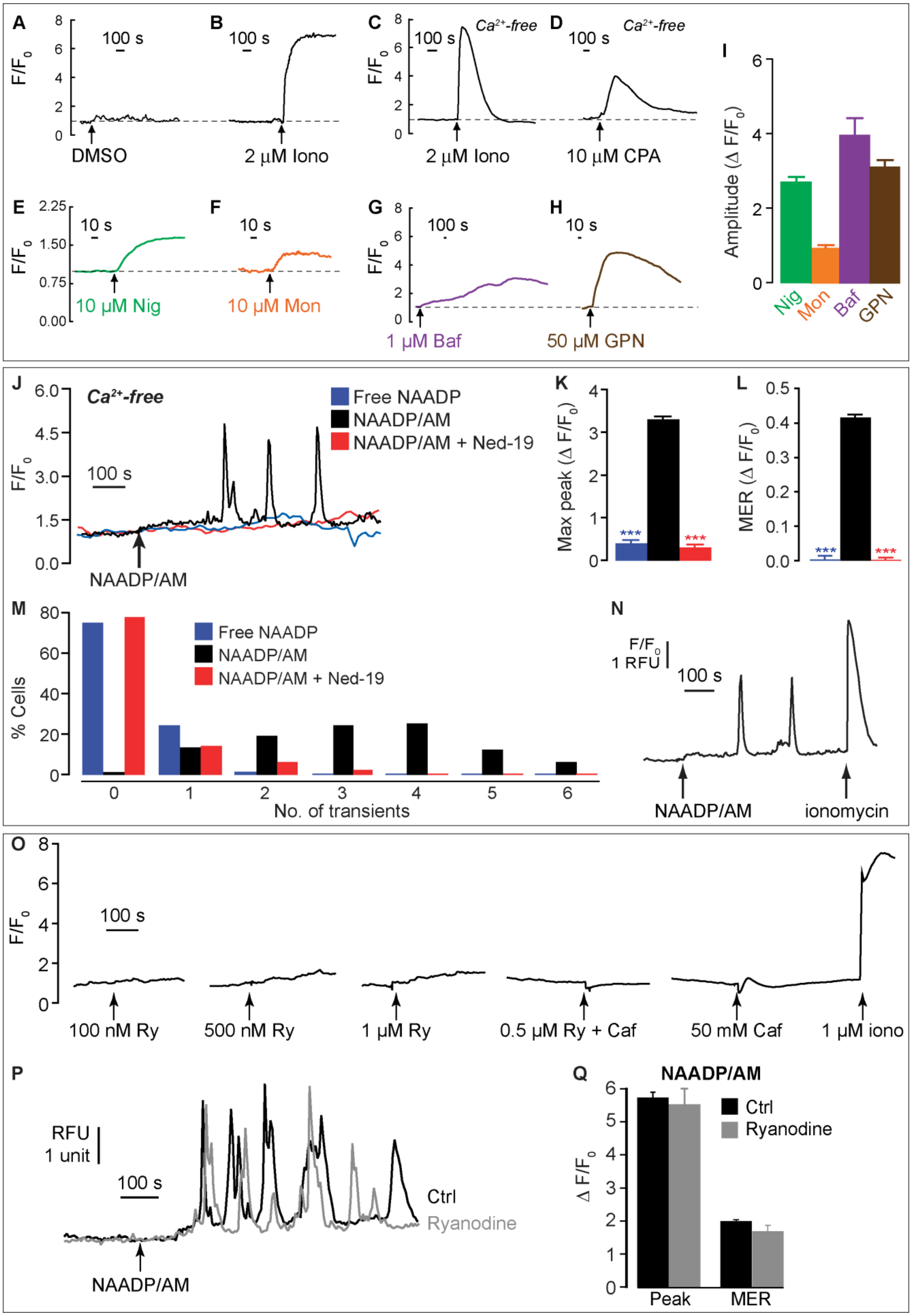

A
B
C
D
I
Ca²⁺-free
Ca²⁺-free
F/F0
100 s
DMSO
2 µM Iono
2 µM Iono
10 µM CPA
Amplitude (Δ F/F0)
E
F
G
H
10 s
100 s
10 µM Nig
10 µM Mon
1 µM Baf
50 µM GPN
Nig
Mon
Baf
GPN
J
K
L
Ca²⁺-free
Free NAADP
NAADP/AM
NAADP/AM + Ned-19
NAADP/AM
Max peak (Δ F/F0)
MER (Δ F/F0)
M
N
% Cells
No. of transients
F/F0
1 RFU
ionomycin
O
100 nM Ry
500 nM Ry
1 µM Ry
0.5 µM Ry + Caf
50 mM Caf
1 µM iono
P
Q
RFU
1 unit
Ctrl
Ryanodine
NAADP/AM
Δ F/F0
Peak
MER

Figure S1.

Figure S1, Related to Figure 1. Ca^{2+} Signals in CTL upon Stimulation with NAADP/AM

(A-H) $[Ca^{2+}]_i$ responses from representative single CTLs normalized to initial fluorescence (F/F_0) upon addition (indicated by arrow) of agents which deplete neutral (ER) (B-D) and acidic (E-H) intracellular Ca^{2+} stores.

(I) Fluorescence changes were determined as the difference between the basal and the maximum fluorescence, $\Delta F/F_0$.

(J-N) $[Ca^{2+}]_i$ changes were monitored in fluo-3-loaded CTLs in 100 μM EGTA-containing Ca^{2+}-free media upon addition of 2.5-10 μM extracellular NAADP/AM.

(J, N) $[Ca^{2+}]_i$ traces are from representative single cells normalized to initial fluorescence (F/F_0). Cells responded to ionomycin (1 μM) at the end of the recording.

(K) Maximal peak fluorescence changes were determined as the difference between basal and the maximum fluorescence, $\Delta F/F_0$.

(L) MER (Mean Elevated Ratio of the mean fluorescence divided by F_0) post-addition of NAADP/AM.

(M) Number of Ca^{2+} transients evoked upon NAADP/AM addition. NAADP/AM elicited Ca^{2+} transients, which were blocked by *trans*-Ned-19 (10 μM). Data are plotted as the mean +/- SEM of 273-730 cells. ***P<0.001 vs NAADP/AM.

(O) Representative traces showing that CTLs did not show any $[Ca^{2+}]_i$ changes in response to ryanodine (Ry) or caffeine (Caf) either in isolation or combined.

(P, Q) 2.5 – 10 μM NAADP/AM responses in CTLs were not affected by pre-incubation with 10 μM ryanodine. n = 99.

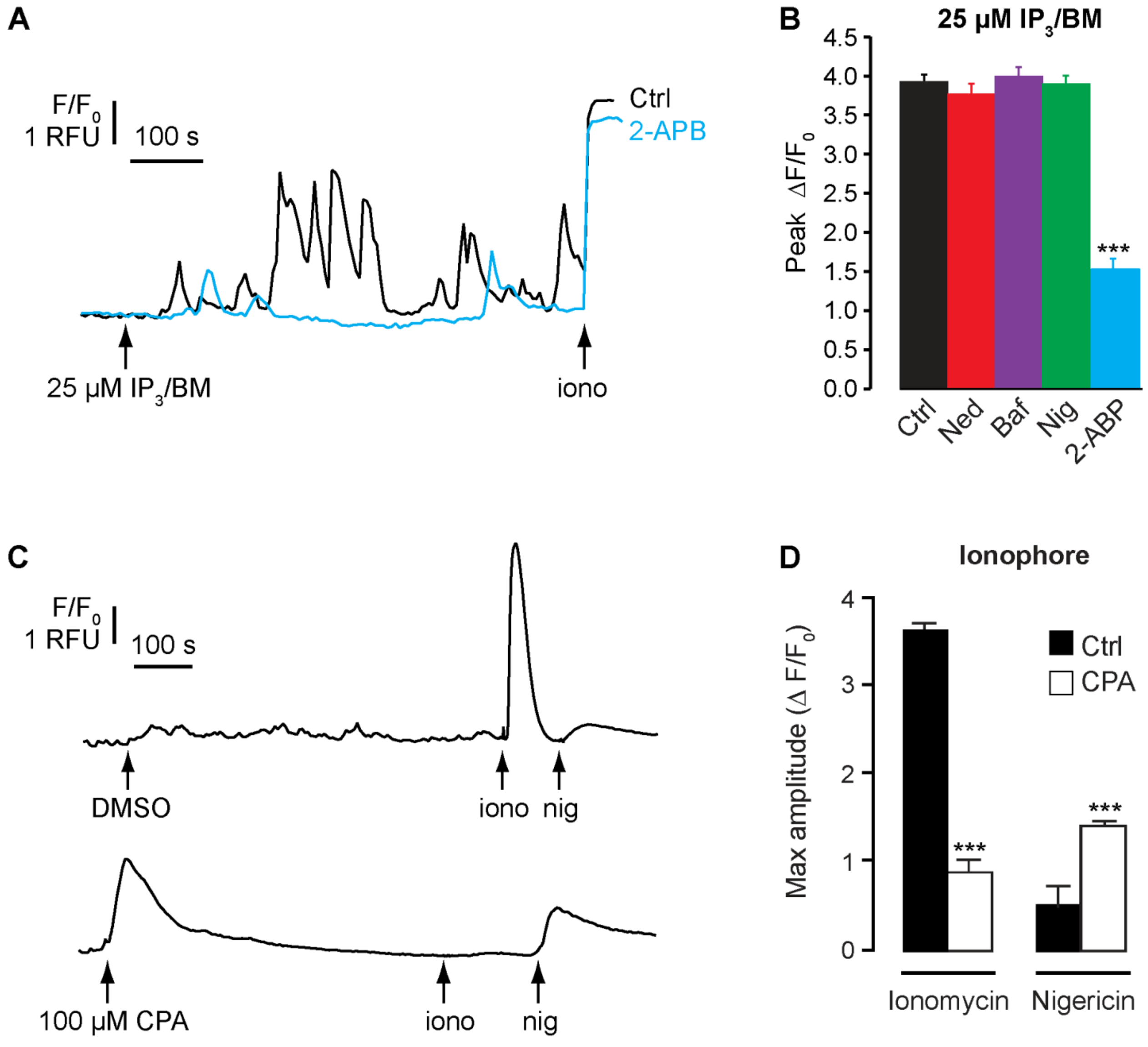

Figure S2, Related to Figure 2. 2-APB Blocks IP_3Rs and CPA Empties ER Ca^{2+} Stores

(A) Representative traces showing Ca^{2+} oscillations monitored in single fluo-3-loaded CTL, elicited by IP_3/BM (25 µM) without (ctrl) or with prior incubation with the IP_3 receptor antagonist 2-ABP (2 µM). Cells responded to ionomycin (1 µM) at the end of the recording.

(B) Fluorescence changes were determined as the difference between basal and the maximum fluorescence, Ca^{2+} release from the ER induced by IP_3/BM (25 µM) was blocked by 2-ABP (2 µM), but not by inhibitors of the NAADP / acidic Ca^{2+} store pathway; *trans* Ned-19 (10 µM), bafilomycin A1 (1 µM) or nigericin (10 µM). Data are plotted as the mean ± SEM of 53-148 cells. ***P<0.001 versus Ctrl.

(C, D) $[Ca^{2+}]_i$ responses from representative single cells normalized to initial fluorescence (F/F_0) in Ca^{2+}-free media containing 100 µM EGTA. ER Ca^{2+} stores were emptied with the SERCA inhibitor, CPA (100 µM), as shown by the lack of Ca^{2+} release by ionomycin (1 µM) following CPA treatment. In contrast, Ca^{2+} released from the acidic Ca^{2+} store (upon addition of 10 µM nigericin) was increased. Data are plotted as the mean +/- SEM of 72-81 cells. ***P<0.001 Ctrl vs CPA.

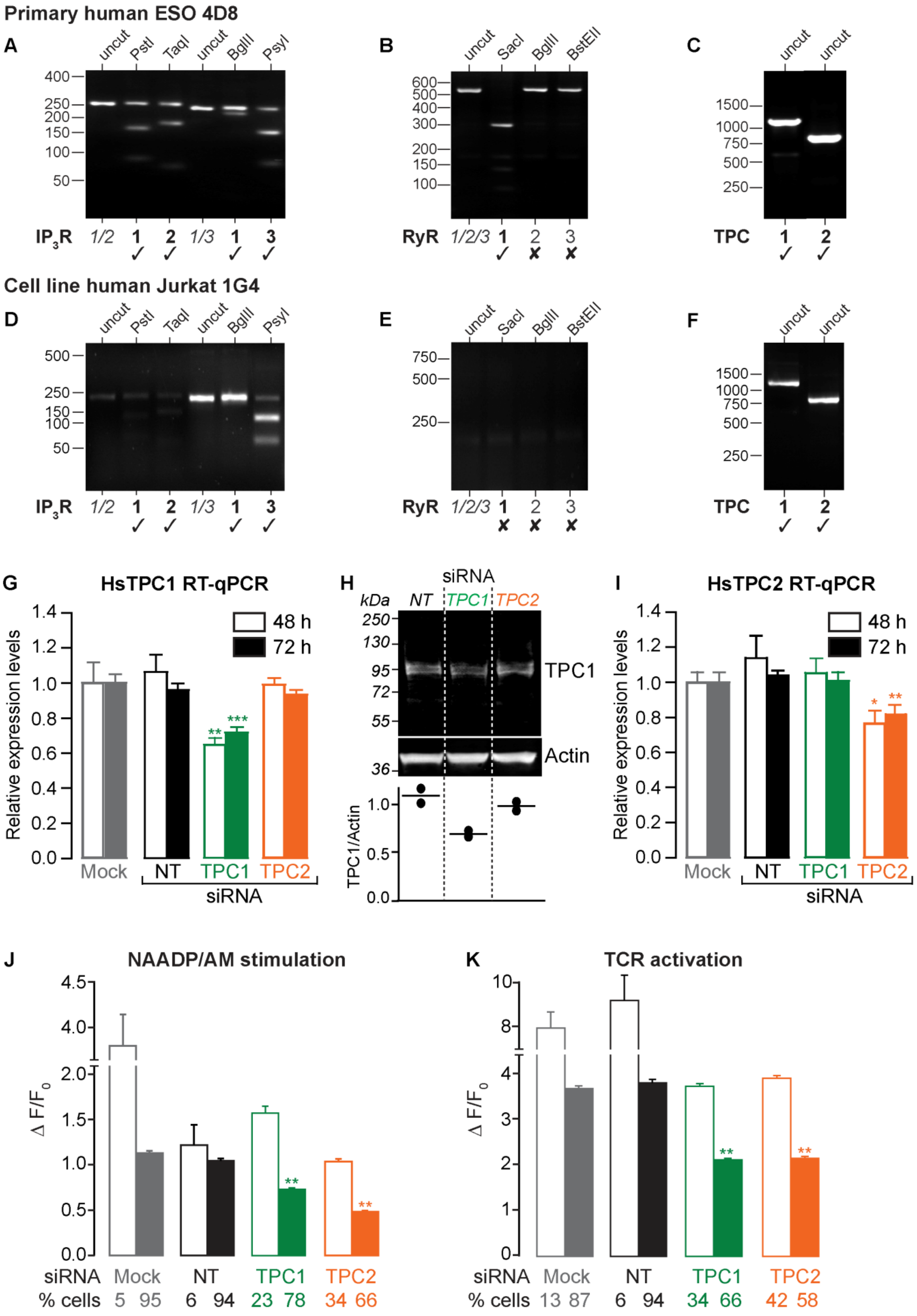

Primary human ESO 4D8
A
uncut PstI TaqI uncut BglII PsyI
IP3R 1/2 1 2 1/3 1 3
B
uncut SacI BglII BstEII
RyR 1/2/3 1 2 3
C
uncut uncut
TPC 1 2
Cell line human Jurkat 1G4
D
uncut PstI TaqI uncut BglII PsyI
IP3R 1/2 1 2 1/3 1 3
E
uncut SacI BglII BstEII
RyR 1/2/3 1 2 3
F
uncut uncut
TPC 1 2
G
HsTPC1 RT-qPCR
48 h
72 h
Relative expression levels
Mock NT TPC1 TPC2
siRNA
H
siRNA
kDa NT TPC1 TPC2
TPC1
Actin
TPC1/Actin
I
HsTPC2 RT-qPCR
48 h
72 h
Relative expression levels
Mock NT TPC1 TPC2
siRNA
J
NAADP/AM stimulation
Δ F/F0
siRNA Mock NT TPC1 TPC2
% cells 5 95 6 94 23 78 34 66
K
TCR activation
Δ F/F0
siRNA Mock NT TPC1 TPC2
% cells 13 87 6 94 34 66 42 58

Figure S3.

Figure S3, Related to Figure 3. TPC Expression Is Reduced by siRNA

(A-F) Intracellular Ca^{2+} channel expression in human ESO 4D8 CTL (A-C) and Jurkat 1G4 cells (D-F).

(A, B, D, E) RT-PCR detection of human IP_3R (A, D) and RyR (B, E) subtypes was performed as described in Supplemental Experimental Procedures using pan primers (uncut). RT-PCR products were digested with restriction enzymes indicated, to give a unique pattern of digestion fragments to identify each isoform, as indicated by a tick if present and a cross if absent. Products and fragments were analysed by electrophoresis in a 2% agarose gel.

(C, F) RT-PCR detection of human TPC1 and 2 were confirmed by DNA sequencing. Representative gels from 3 independent RT-PCR reactions are shown.

(G-K) Jurkat 1G4 cells were nucleofected with 100 pmol siRNA (non-targeting (NT), TPC1 and TPC2) and incubated for 48-72 h. Mock indicates cells that had been nucleofected, but were without siRNA.

(G, I) Total RNA was isolated and RT-qPCR was conducted, as detailed in Supplemental Experimental Procedures. Human TPC1 (G) and TPC2 (I) showed isoform-specific reduction of mRNA levels by knockdown using siRNA (n = 6). Data is expressed as relative expression levels (arbituary units) calculated from the normalization of the mean ΔCp value to the geometric mean of 3 reference genes ± S.E.M. *P<0.05, **P<0.01, ***P<0.001 vs Mock control.

(H) Western blot of endogenous HsTPC1 protein in Jurkat 1G4 cells (predicted molecular weight of 94.1 kDa). TPC1-targeted siRNA specifically reduced TPC1 protein by ~32% as normalized to the actin control.

(J, K) siRNA-treated cells were stimulated with 10 µM NAADP/AM (J) or by contact with 10 µg/ml anti-human CD3-coated coverslips (K). Summary of first peak Ca^{2+} changes, n = 213-383 cells from 6 independent siRNA experiments. For each condition, single-cell responses were categorized into two sub-populations based on k-means cluster analysis; the mean responses for each cluster were plotted separately. The number of cells within each population is indicated under each bar as a percentage of the total cell number. **P<0.01 versus NT cluster containing the highest percentage of cells.

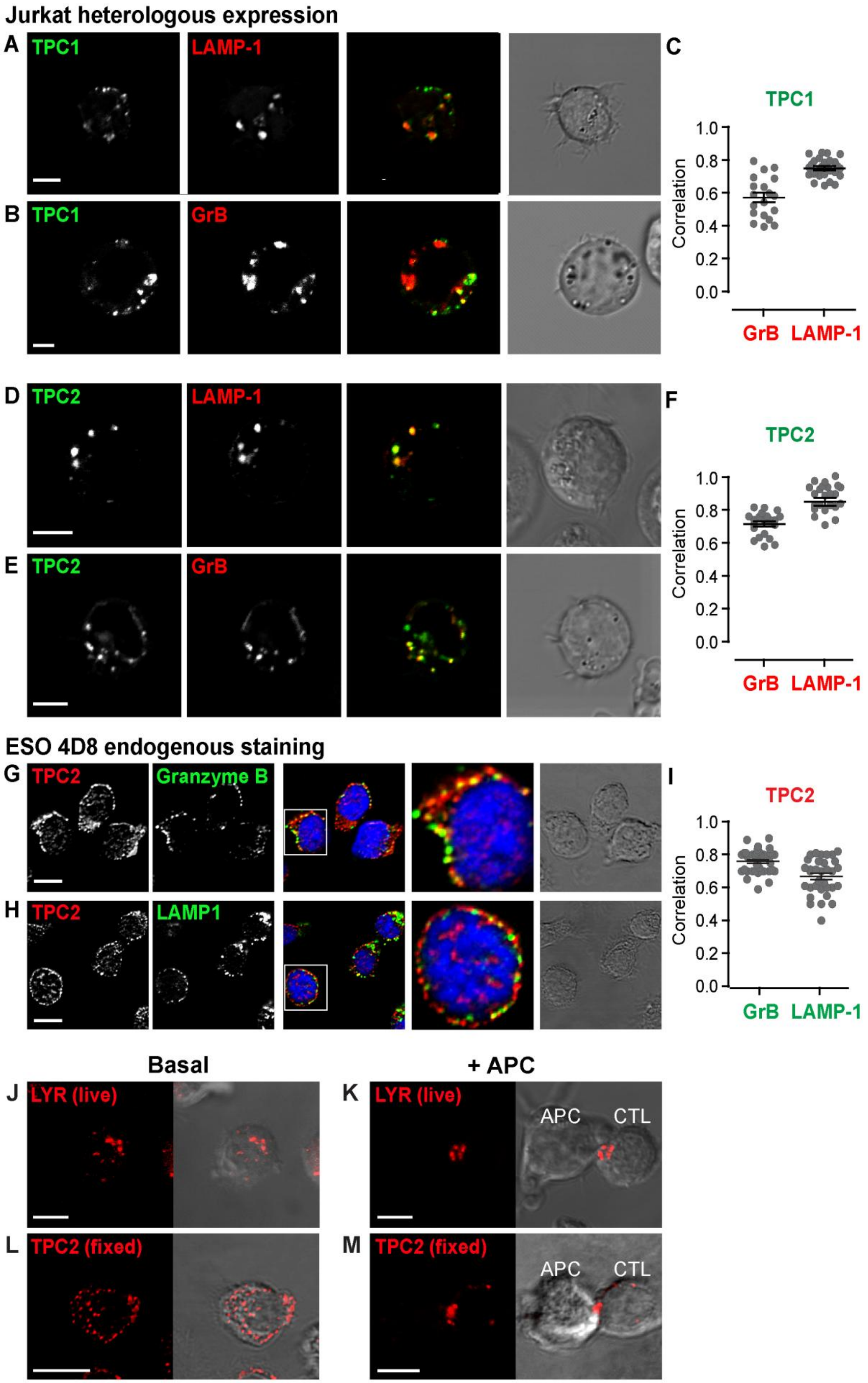
Jurkat heterologous expression
A
TPC1
LAMP-1
B
TPC1
GrB
C
TPC1
1.0
0.8
0.6
0.4
0.2
0.0
Correlation
GrB
LAMP-1
D
TPC2
LAMP-1
E
TPC2
GrB
F
TPC2
1.0
0.8
0.6
0.4
0.2
0.0
Correlation
GrB
LAMP-1
ESO 4D8 endogenous staining
G
TPC2
Granzyme B
H
TPC2
LAMP1
I
TPC2
1.0
0.8
0.6
0.4
0.2
0.0
Correlation
GrB
LAMP-1
Basal
+ APC
J
LYR (live)
K
LYR (live)
APC
CTL
L
TPC2 (fixed)
M
TPC2 (fixed)
APC
CTL

Figure S4.

Figure S4, Related to Figure 4. TPCs Are Localized on Exocytotic (Cytolytic) Granules

(A-I) Colocalization of TPCs with cytolytic granule markers.

(A, B, D, E) Representative confocal microscope images from Jurkat 1G4 cells transiently coexpressing human TPC1 (A, B, in green) or human TPC2 (D, E, in green) tagged at the C-terminus with mCherry or eYFP with human LAMP1–mGFP (A, D, in red) or human Granzyme B–RFP (B, E, in red).

(C, F, I) The overlay images give an indication as to the extent of colocalization (shown in yellow) and quantified by the Mander's correlation coefficient where values closer to 1.0 indicate greater colocalization (n = 28-39 cells).

(G, H) In ESO 4D8 primary CTLs endogenous human TPC2 (in red) was labelled with an in-house anti-human TPC2 antibody (specificity was confirmed by significant peptide block: fluorescence units 1332 ± 36 for anti-TPC2 versus 167 ± 5 (mean ± SEM) for peptide block, n = 149-158 cells). Fixed samples were costained for the cytolytic granule granzyme B and LAMP-1 (in green) and the nucleus stained with Hoechst (in blue). Scale bar corresponds to 5 µm.

(J-M) Confocal images of ESO 4D8 CTL loaded with (J, K) Lysotracker Red to label acidic cytolytic granules (live cells) or (L, M) immunolabeling of endogenous TPC2 (fixed cells) before (basal) and after engagement with Antigen-Presenting Cells (+APC). Lysotracker Red and TPC2 labeling move to the immunological synapse – the contact point between the CTL and its target cell.

Supplemental Experimental Procedures

Cells and Reagents

NY-ESO-1 specific 4D8 CTL clone was sorted directly using HLA-A2 NY-ESO-$1_{157\text{-}165}$ tetramers from a melanoma patient, following a protocol as described in [1]. After expansion, CTLs were stimulated with irradiated feeder in the presence of 5 µg/ml phytohaemagglutinin and cultured over 12 days at 37°C, 5% CO_2 in RPMI-1640 (Invitrogen) supplemented with 10% (v/v) foetal calf serum, 2 mM Glutamax, 50-100 U/ml penicillin and 100 µg/ml streptomycin, 10 mM HEPES, 50 µM 2-mercaptoethanol, 1 mM sodium pyruvate, non-essential amino acids and 25 U/ml recombinant human IL-2 (Roche). Jurkat 1G4 cells were generated by lentiviral transduction of the 1G4 TCR [1] into Jurkat J.RT3-T3.5 cells and were maintained in RPMI-1640 supplemented with 10% (v/v) foetal calf serum, 2 mM Glutamax, 50-100 U/ml penicillin and 100 µg/ml streptomycin.

NAADP/AM [2] and IP_3/BM [3] were synthesized in-house. *Trans*-Ned-19 was purchased from Enzo Life Sciences; Ionomycin and Cyclopiazonic Acid were from Calbiochem; Nigericin, monensin, GPN (glycyl-phenylalanine 2-naphthylamide), 2-APB (2-aminoxydiphenylborate) and phorbol myristate acetate (PMA) were from Sigma-Aldrich and Bafilomycin A1 was purchased from LC laboratories. Fluo-3/AM, Pluronic F-127 and FM 4-64 were from Invitrogen. NY-ESO-1 9C peptide [1] was treated with 20 mM TCEP (1h at 37°C) before being used at a final concentration of 100 nM. The LAMP1-mGFP plasmid [4] was a gift from E.C. Dell'Angelica (University of California, Los Angeles) and the DsRed-human Granzyme B plasmid [5] was provided by E.O. Long (NIH).

Intracellular Ca^{2+} Measurements

Ca^{2+} was measured in the ESO 4D8 CTL clone and Jurkat 1G4 cells by laser scanning confocal microscopy. Cells adhering to poly-lysine-coated glass coverslips were loaded with 5 µM fluo-3/AM (Invitrogen) plus 0.03% (w/v) Pluronic F127 in RPMI 1640 for 30 min at room temperature and mounted on the stage of a Zeiss LSM510 Meta confocal laser-scanning microscope (Ex 488 nm, Em > 505 nm) equipped with a 63x objective. After loading, cells were either washed in Ca^{2+}-containing RPMI-1640 (phenol red-free) or in Ca^{2+}-free phosphate-buffered saline (PBS) containing 5 mM glucose, 0.5 mM $MgCl_2$ and 1 mM EGTA at pH 7.4 (EGTA was reduced to 100 µM for experiments). *For antigen presentation experiments*, T2 target cells were pulsed with 100 nM NY-ESO-$1_{157\text{-}165}$ peptide (SLLMWITQC) for 90 min at 37°C and washed twice with FCS-free RPMI 1640 medium, and then added to the cell chamber and allowed to engage with ESO 4D8 CTLs under gravity. Experiments were conducted at room temperature with an image collected every 1-5 s. Similar results were observed with fura-2 and at 37°C [1]. The fluorescence of single cells was measured and expressed as fold changes over basal (F/F_0).

Human Granzyme B ELISA

CTLs and peptide-pulsed (100 nM ESO 9C) T2 target cells were incubated at 37°C for 15 min at an E:T ratio of 1:1, and the supernatant harvested and assayed for Granzyme B (GrB) by ELISA (Mabtech). Briefly, 96-well high-protein binding ELISA plates (Greiner) were coated overnight at 4°C with 2 µg/ml mouse anti-human GrB mAb (clone GB10) in PBS, pH 7.4. Following washes with PBS-Tween 20 (0.05%) and incubation with a 0.1% BSA block, GrB standards and samples were added to wells and incubated for 2h at room temperature. Biotinylated mouse anti-human GrB (clone GB11) at 1 µg/ml in 0.1% BSA/PBS solution was added to wells for 1 h then washed 5x with PBS-Tween. Streptavidin-alkaline phosphatase, diluted 1:1000 in 0.1% BSA/PBS, was added for 1 h. ELISA plates were washed and GrB was detected upon addition of substrate Di(Tris) *p*-nitrophenyl phosphate (Sigma) and absorbance was measured at 405 nm.

^{51}Cr-Release Assay

4D8 CTLs were pre-incubated either with 10 µM *trans*-Ned-19, 1 µM Bafilomycin A1, 2 or 50 µM 2-APB or 0.1% DMSO for 30 min and the experiment conducted in the continual presence of the drug. Cytotoxicity was assessed using T2 target cells loaded with 100 nM ESO 9C peptide and labelled with ^{51}Cr for 90 min at 37°C, cells were washed three times. 4D8 CTL were cocultured with T2 target cells at the indicated cell ratio. ^{51}Cr release was measured after incubation with CTL for 2 h at 37°C. The percentage of specific lysis was calculated as: 100 × [(experimental – spontaneous release)/(total – spontaneous release)].

Reverse Transcription and PCR Amplification (RT-PCR)
Total RNA was isolated from $2.5x10^5$ ESO 4D8 cells or Jurkat 1G4 cells using a RNeasy mini kit (Qiagen) and DNaseI treated. RT-PCR amplification of human TPC, RyR and IP_3 receptors was conducted using the Superscript III one-step RT-PCR kit (Invitrogen). Briefly, 100 ng of total RNA was incubated with 0.2 µM of forward and reverse primers (custom-synthesized by Invitrogen) and the superscript III RT/Taq mix. The oligonucleotide primers (5'-3') and protocols used for amplification of the first strand cDNA (given in brackets) are as follows:

TPCN1 – CCTCCAGGAAGGCGAGAACA and CGTTGACTGCCACCACCAAG (94–61.5–68°C/15–30–90 s/40 cycles);

TPCN2 – AGTTCCTCTTCGGCCACTACT and CCAGCCACCACAATACAAAA (94–58.5–68°C/15–30–90 s/40 cycles);

RyR – CA(C/T)(C/T)T(A/C/G/T)(C/T)T(A/C/G/T)GA(C/T)AT(A/C/T)GCIATGGG and A(A/G/T)(A/G)TA(A/G)TT(A/C/G/T)GCIA(A/G)(A/G)TT(A/G)TG(C/T)TC (94–52–68°C/15–30–60 s/40 cycles) [6];

IP_3R 1/2 – CTTCATC(A)GTCCTGGTGAAAGT and CTTCCTTTGTTCTGTCATCTG; *IP_3R 1/3* – AGTGAGAAGCAGAAGAAGG and CATCC(G)TGGGGAACCAGTC (94–52–68°C/15–30–60 s/40 cycles) [7].

Restriction Enzyme Analysis of RT-PCR Products
Since degenerate primers were used to amplify RyR and IP_3R, RT-PCR products were subjected to restriction enzyme analysis to determine which isoforms were present. For RyR analysis three restriction enzymes (Fermentas) were used: *Sac*I, *Bgl*II and *Bst*EII; *Sac*I cut RyR1 only into 300-, 130- and 100-bp fragments, *Bgl*II cut RyR2 only into 295- and 235-bp fragments and *Bst*EII cut RyR3 only into 280- and 250-bp fragments [6]. For IP_3R analysis, *Pst*I and *Taq*I distinguished between IP_3R1 (168- and 88-bp fragments) and IP_3R2 (182- and 74-bp fragments) respectively. Whilst *Bgl*II and *Asp*I distinguished IP_3R1 (220- and 23-bp fragments) from IP_3R3 (159- and 84-bp fragments) respectively. TPC expression was confirmed by DNA sequencing (GATC Biotech) of RT-PCR products.

Nucleofection of Jurkat 1G4 with Plasmid DNA and siRNA
1×10^6 Jurkat 1G4 cells were suspended in 100 µl of T cell Nucleofector solution (Amaxa cell line kit V, Lonza) and mixed with 100 pmol siRNA or 5 µg of plasmid DNA. Cells were then placed in a cuvette and given an electric pulse (program X-001 or X-005 for siRNA or plasmid nucleofection, respectively) using a Nucleofector device II (Amaxa). Cells were resuspended in 1.5 ml of RPMI-1640 medium supplemented with 10% FCS, 50-100 U/ml penicillin, 100 µg/ml streptomycin and L-glutamine and incubated in 12-well plates at 37°C, 5% CO_2. 1 ml of media was replaced 4-5 h post-nucleofection. The following ON-TARGET*plus* siRNA were purchased from Dharmacon: non-targeting pool, human *TPCN1* SMART pool and human *TPCN2* SMART pool.

RT-qPCR
RNA was isolated from Jurkat 1G4 cells treated with siRNA, using a RNeasy micro kit (Qiagen) and DNase I treated, as per manufacturer's instructions. cDNA was synthesized from 40 ng/µl of total RNA and random primers using a high-capacity cDNA reverse transcription kit (Applied Biosystems) following the manufacturer's instructions. For qPCR, 5 ng/µl cDNA, 0.1 µM forward and reverse primers, and their corresponding universal probe (Roche) were mixed with the LightCycler® 480 Probes Master (Roche Applied Science). The following human primer (5'-3') and probe sets were designed using the Roche™ Universal Probe Library Assay Design Center:

TPCN1 – ACTCCGGCTTGGCATCTAT and ACTACCACCATCAGGGCAAA with probe #1;
TPCN2 – GGCCACCTACCAGATGACTG and CTCTGTGAGCTCATCCTCCTC with probe #25;
GAPDH – ACACCCACTCCTCCACCTTT and TGACAAAGTGGTCGTTGAGG with probe #45;
β-Actin – CCAACCGCGAGAAGATGA and TCCATCACGATGCCAGTG with probe #64;
PPIB – TGTGGTGTTTGGCAAAGTTC and GTTTATCCCGGCTGTCTGTC with probe #10.

Each qPCR reaction was performed in duplicate on a LightCycler® 480 Instrument (Roche Applied Science). The cycling conditions comprised 50°C (2 min), 95°C (10 min) and 40 cycles at 95°C (15 s) and 60°C (1 min), followed by 40°C (30 s). Samples were analysed by the LightCycler® 480 system to attain Cp values. Standard curves were generated from serially diluted pooled cDNA from all samples analysed and used to determine the amplification efficiency (E) for each gene analysed – all PCR efficiencies were

above 95%. Relative (ΔCp) values for each gene set were calculated by applying the formula $E^{(\text{lowest Cp-Cp})}$. Finally, the ΔCp for *TPCN1* or *TPCN2* were normalised to the geometric mean of three reference genes (GAPDH, β-Actin and PPIB), expressed in arbitrary units. geNorm was used to determine stable expression of the three reference genes used [8].

Immunoblotting

Pellets of Jurkat 1G4 cells were resuspended in 20 mM HEPES, 1 mM EDTA (pH 7.2) supplemented with EDTA-free protease inhibitor mix (Roche) and homogenized in a glass dounce. Nuclei and unbroken cells were removed by centrifugation at 1500 g for 3 min. Proteins (40 µg/lane) were resolved by SDS-PAGE using 8% tris-glycine gels (NuSep) and transferred to PVDF membranes. Membranes were blocked in Odyssey blocking buffer (LI-COR) and incubated with goat anti-TPC1 antibody (Santa Cruz) and mouse anti-β-actin (abcam) in Odyssey buffer and 0.1% Tween-20. Secondary antibodies were anti-goat and anti-mouse IgGs conjugated to Alexa-fluors (Invitrogen). Immunoreactive bands were detected by fluorescence using a Typhoon scanner (GE Healthcare). To minimize heat-induced aggregation, protein samples loaded on gels contained 200 µM DTT and were not heat-treated.

Immunolocalization

ESO 4D8 CTLs were fixed with 4% paraformaldehyde and permeablized with 0.2% Triton X-100. After blocking sites with 2% goat serum and 0.5% foetal bovine serum, samples were incubated with 10 µg/ml anti-human TPC2 antibody (in-house peptide antibody, affinity-purified), 1 µg/ml anti-Granzyme B (clone GB11, Mabtech) or 1 µg/ml anti-LAMP1 (clone H4A3, BD Biosciences). Specificity of anti-TPC2 labeling was determined using 100 µg/ml of competing immunogenic peptide. Labeling was detected with Alexa-Fluor secondary antibodies (Invitrogen). Immunofluorescence was viewed on a Zeiss 510 Meta confocal laser scanning microscope and images collected in the Multitrack mode which alternates laser lines to reduce bleed through. Excitation/emission (nm): green (488/505-530), red (543/>560).

TPC Translocation to the IS

A pseudoimmunological synapse can be formed between T-cells and glass coverslips coated with αCD3 antibody [9]. Therefore, Jurkat cells expressing C-terminus YFP-tagged TPC1 or TPC2 were seeded onto coverslips either with or without antibody coating and imaged 15-60 min after adhering. In some experiments, cells were allowed to adhere and then incubated for 10-15 min with the lipophilic dye FM 6-64 (5 µM) to label the plasma membrane. Chambers were maintained at room temperature and mounted on the stage of a Nikon A1 confocal laser scanning microscope equipped with a 60x (NA 1.49) objective. Excitation/emission wavelengths were 488/500-550 nm (YFP) and 561/570-620 nm (FM 4-64) using a 0.44-µm optical slice thickness. Single-cell Z-stacks were collected using a 0.38-µm slice interval over a 9-13 µm depth range. TPC-YFP was quantified in each slice as the product of the fluorescence intensity and its area using thresholding in Nikon Elements software and data were plotted as the product (as a percent of the cell total) versus cell depth. Our quantification of TPC density does not take into account vesicle clustering towards the cell centre and is, therefore, an underestimate of the polarization.

Data Analysis

Data are presented as the mean ± SEM of n cells.

To quantitatively assess whether only a sub-population of cells were affected by siRNA, we used a k-means clustering method (SPSS Statistics software, IBM). Ca^{2+} data from individual single cells were distributed into 2 groups by performing an iterative algorithm to reduce intra-cluster variability whilst increasing inter-group variability. $\Delta F/F_0$ values were categorized into 2 clusters (sub-populations) and each cluster expressed as the mean ± SEM. Control groups (Mock and NT) were essentially sorted into 1 cluster (6-13% of cells were outliers). The number of cells assigned to a given cluster were expressed as the percentage of the total number of cells.

Statistical analysis was performed using Student's t test for two data sets, whereas multiple groups were analysed using ANOVA and a Tukey-Kramer or Dunnett's post-test — significance (*) was assumed at *P < 0.05, **P < 0.01, ***P < 0.001.

Supplemental References

1. Chen, J.L., Morgan, A.J., Stewart-Jones, G., Shepherd, D., Bossi, G., Wooldridge, L., Hutchinson, S.L., Sewell, A.K., Griffiths, G.M., van der Merwe, P.A., et al. (2010). Ca2+ release from the endoplasmic reticulum of NY-ESO-1-specific T cells is modulated by the affinity of TCR and by the use of the CD8 coreceptor. J Immunol *184*, 1829-1839.
2. Parkesh, R., Lewis, A.M., Aley, P.K., Arredouani, A., Rossi, S., Tavares, R., Vasudevan, S.R., Rosen, D., Galione, A., Dowden, J., et al. (2008). Cell-permeant NAADP: a novel chemical tool enabling the study of Ca2+ signalling in intact cells. Cell Calcium *43*, 531-538.
3. Conway, S.J., Thuring, J.W., Andreu, S., Kvinlaug, B.T., Roderick, H.L., Bootman, M.D., and Holmes, A.B. (2006). The synthesis of membrane permeant derivatives of *myo*-inositol 1,4,5-trisphosphate. Aust J Chem *59*, 887-893.
4. Falcon-Perez, J.M., Nazarian, R., Sabatti, C., and Dell'Angelica, E.C. (2005). Distribution and dynamics of Lamp1-containing endocytic organelles in fibroblasts deficient in BLOC-3. J Cell Sci *118*, 5243-5255.
5. Liu, D., Martina, J.A., Wu, X.S., Hammer, J.A., 3rd, and Long, E.O. (2011). Two modes of lytic granule fusion during degranulation by natural killer cells. Immunol Cell Biol *89*, 728-738.
6. Bennett, D.L., Cheek, T.R., Berridge, M.J., De Smedt, H., Parys, J.B., Missiaen, L., and Bootman, M.D. (1996). Expression and function of ryanodine receptors in nonexcitable cells. J Biol Chem *271*, 6356-6362.
7. Morgan, J.M., De Smedt, H., and Gillespie, J.I. (1996). Identification of three isoforms of the $InsP_3$ receptor in human myometrial smooth muscle. Pflugers Arch *431*, 697-705.
8. Vandesompele, J., De Preter, K., Pattyn, F., Poppe, B., Van Roy, N., De Paepe, A., and Speleman, F. (2002). Accurate normalization of real-time quantitative RT-PCR data by geometric averaging of multiple internal control genes. Genome Biol *3*, 1-12.
9. Balagopalan, L., Sherman, E., Barr, V.A., and Samelson, L.E. (2011). Imaging techniques for assaying lymphocyte activation in action. Nat Rev Immunol *11*, 21-33.
